# Enhancing Absorption Performance of CO_2_ by Amine Solution through the Spiral Wired Channel in Concentric Circular Membrane Contactors

**DOI:** 10.3390/membranes12010004

**Published:** 2021-12-21

**Authors:** Chii-Dong Ho, Hsuan Chang, Guan-Hong Lin, Thiam Leng Chew

**Affiliations:** 1 Department of Chemical and Materials Engineering, Tamkang University, Tamsui, New Taipei 251, Taiwan; nhchang@mail.tku.edu.tw (H.C.); wesleycan199@gmail.com (G.-H.L.); 2 Department of Chemical Engineering, Faculty of Engineering, Universiti Teknologi Petronas, Seri Iskandar 32610, Malaysia; thiamleng.chew@utp.edu.my; 3 CO_2 _Research Centre (COSRES), Institute of Contaminant Management, Universiti Teknologi PETRONAS, Seri Iskandar 32610, Malaysia

**Keywords:** spiral wired annulus channel, carbon dioxide absorption, sherwood number, concentric-tube membrane contactor, concentration polarization

## Abstract

The CO_2_ absorption rate by using a Monoethanolamide (MEA) solution through the spiral wired channel in concentric circular membrane contactors under both concurrent-flow and countercurrent-flow operations was investigated experimentally and theoretically. The one-dimensional mathematical modeling equation developed for predicting the absorption rate and concentration distributions was solved numerically using the fourth Runge–Kutta method under various absorbent flow rate, CO_2_ feed flow rate and inlet CO_2_ concentration in the gas feed. An economical viewpoint of the spiral wired module was examined by assessing both absorption flux improvement and power consumption increment. Meanwhile, the correlated average Sherwood number to predict the mass-transfer coefficient of the CO_2_ absorption mechanisms in a concentric circular membrane contactor with the spiral wired annulus channel is also obtained in a generalized and simplified expression. The theoretical predictions of absorption flux improvement were validated by experimental results in good agreements. The amine solution flowing through the annulus of a concentric circular tube, which was inserted in a tight-fitting spiral wire in a small annular spacing, could enhance the CO_2_ absorption flux improvement due to reduction of the concentration polarization effect. A larger concentration polarization coefficient (CPC) was achieved in the countercurrent-flow operations than that in concurrent-flow operations for various operations conditions and spiral-wire pitches. The absorption flux improvement for inserting spiral wire in the concentric circular module could provide the maximum relative increment up to 46.45%.

## 1. Introduction

The accelerated industrial movement development during the last few decades results in increasing flue gases from fossil fuel combustion containing CO_2_ in greenhouse gas emission, which speeded the environmental concerns [1] in global warming issues. Meanwhile, the biogas is processed and conditioned by removing impurities such as CO_2_ (30–45%) and H_2_S (0.5–1%) to upgrade its value and satisfy pipeline transport specifications. CO_2_ capture using several technologies, namely absorption [2], adsorption [3], and membrane processes [4] of which the membrane contactor is a promising alternative technology with high absorption efficiency due to offering the advantages of low energy consumption, the independent control of gas and absorbent flow rates, a large mass-transfer area, continuous operations, and the flexibility to scale up [5]. Either physical or chemical absorption is the most common purification technology for gas separation for all these applications, especially for combining both chemical absorption and the separation technique [6] to allow the soluble gas mixture components to be selectively absorbed on the membrane surface of the liquid phase in liquid/liquid and gas/liquid systems [7,8].

Successful intensifications of gas/liquid membrane contactors have been developed and employed providing the guideline to the judicious choice of membrane materials [9] and absorbents for CO_2_ absorption processes [10,11]. Previous studies proved some durable and reusable materials used for the membrane contactor of CO_2_ absorption, where the as-prepared hydrophobic polymethylsilsesquioxane (PMSQ) aerogels [12], and hybrid bis(trimethoxysilyl)hexane (BTMSH)/tetraethyl orthosilicate (TEOS) silica aerogels [13] and highly porous polyvinylidene fluoride (PVDF) [14] were used as a membrane contactor indicating a decrease in the mass-transfer resistance for CO_2_ absorption performance. Moreover, the separation efficiency of membrane gas absorption depends on the distribution coefficient and a composition gradient of gas solute in the gas/liquid system [15]. A gas/liquid interface was formed in the pore entrance near the microporous hydrophobic membrane surface of the shell side when the membrane pores are not wetted [16]. It is crucial to develop an effective strategy to capture CO_2_ with the minimum cost [17]. Numerous absorbents in hollow fiber membrane contactors [18,19] were conducted with the lower membrane wettability like amine solution and the properties of absorbents [20] for CO_2_ absorption improvement were further investigated. Karror and Sirkaras [21] investigated a series of comprehensive experiments of gas/liquid absorption in a shell and tube membrane contactors when considering a laminar flow velocity of liquid profile, while Bakhshali et al. [22] employed computational fluid dynamics to show the high efficient removal efficiency of CO_2_ in turbulent flow conditions. Knudsen-molecular diffusion transition models [23], as referred to the dusty gas model, were widely used to describe the mass-transfer behaviors across membranes, and were successfully applied to express the absorption flux performance [24,25].

Membrane separation processes are still facing the problems of the concentration polarization effect despite major advances in developing membrane contactors on gas absorption. The concentration polarization effect building up concentration gradients can be the cause of a considerable reduction in mass-transfer rate [26], which accumulates the retained species and depletes the permeate component in the mass-transfer boundary layer adjacent to the membrane surface, and thus the separation efficiency and permeate flux were decreased [27]. Proposing a prospective strategy [28] included breaking down the laminar sublayer in a turbulent boundary layer region adjacent to the membrane surface by embedding spiral wires into the flowing channel. Hosseinzadeh et al. [29] investigated how absorption efficiency in a parallel-plate gas/liquid polytetrafluoroethylene (PTFE) membrane contactor was augmented by inserting turbulent promoters. The present work focuses on the overall mass-transfer resistance in which the potential investigation of different spiral-wire pitches boost turbulent intensity due to dynamical changing the mass-transfer boundary layer and mitigating concentration polarization. The concentration polarization effect in membrane separation processes plays a vital role in diminishing trans-membrane mass flux in the majority of membrane separation processes, such as gas absorption [30], reverse osmosis [31], extraction [32], pervaporation [33] and dialysis [34]. Various approaches provided a remarkable advantage to minimize the concentration polarization effect for achieving higher mass-transfer rates using eddy promoters [35], such as net spacer channels [36] and carbon-fiber spacer channels [37], where the turbulent intensity enhancement is effectively raised to come out with a higher convective mass-transfer coefficient [38].

The present study develops the mathematical modeling of CO_2_ absorption by using an MEA solution flowing in the lumen of spiral wired concentric-tube module to generate vortices, while the gas mixture CO_2_/N_2_ flows in the tube side. The characteristics of CO_2_ absorption in the MEA solution was investigated in the previous research [39], and the performance improvement of a rotated wired concentric-tube channel was validated for enrichment of heavy water [40]. Theoretical and computational studies were performed for comparisons under various operating conditions to model the CO_2_ absorption process associated with occurring reactions [41] by using amines and mixed amines [42], and to enhance CO_2_ capture efficiency and reduce regeneration cost [43]. The objective of this study is to implement the spiral wires and stick them onto the membrane surface of the flow channel to enhance the local shear stress on the membrane surface and to create secondary flows or eddies in the feed stream, and thus achieve a higher CO_2_ absorption rate. In the present study, the device performance was further improved by inserting various spiral-wire pitches along the flow channel. The helical wire on the circumference of the concentric-tube provided a larger convective mass-transfer coefficient, which disrupted the boundary layer to reduce the mass-transfer resistance, where a higher CO_2_ absorption rate was thus observed. The turbulence intensity induced by embedding spiral wires in the MEA absorbent flow channel was examined by incorporating and regressing a correlated expression of the convective mass-transfer coefficient for the spiral wired concentric-tube membrane contactor. The effects of spiral-wire pitch, MEA feed concentration, and gas and liquid feed flow rates on the absorption flux of CO_2_ were evaluated once the simplified expression was obtained. The trade-off between the CO_2_ absorption flux improvement and energy consumption increment was analyzed in finding the economic assessment in module designs and system operations, and hence the application of the inserting helical wires in the flow channel to design membrane gas absorption modules is technically and economically feasible. Therefore, the absorption mechanisms were studied in the one-dimensional steady-state modeling equation of the mass-balance and chemical reaction, which was developed and simulated theoretically and carried out experimentally on a spiral wired concentric circular module with the use of the PTFE membrane.

## 2. Theoretical Formulation

### 2.1. Mass Transfer

**Figure 1:**
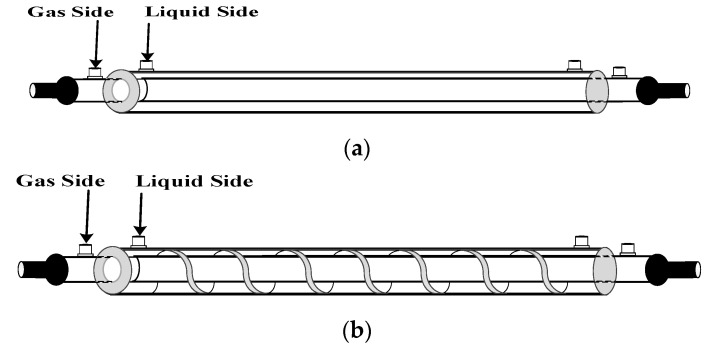
Empty and spiral wired annulus channels of concentric circular membrane contactors. (**a**) Empty channel; (**b**) Spiral wired channel.

**Figure 2:**
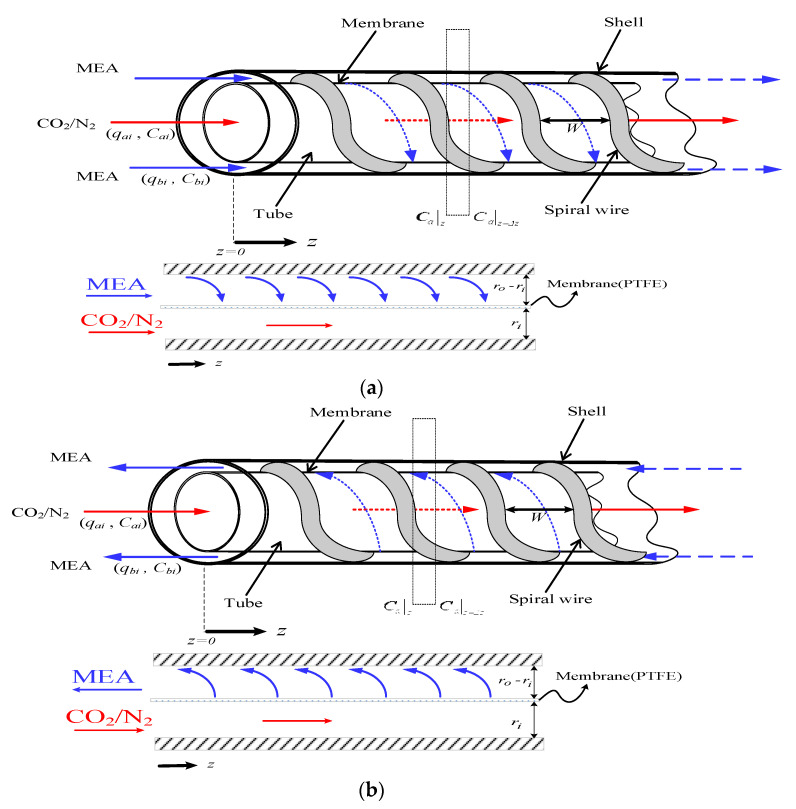
Spiral wired concentric circular membrane contactors. (**a**) Concurrent-flow operations; (**b**) Countercurrent-flow operations.

A concentric circular membrane contactor without/with embedding spiral wires onto the lumen side was fabricated to conduct the experimental work in aiming to enhance the CO_2_ absorption rate by using amine solution, as shown in Figure 1, respectively, while Figure 2 shows schematic representations of both concurrent- and countercurrent-flow operations. Two spiral-wire pitches (2 mm and 3 mm) were embedded into flow channels in comparisons of the device performance with a spiral wired annulus channel and empty channel (without embedding spiral wires).

**Figure 3:**
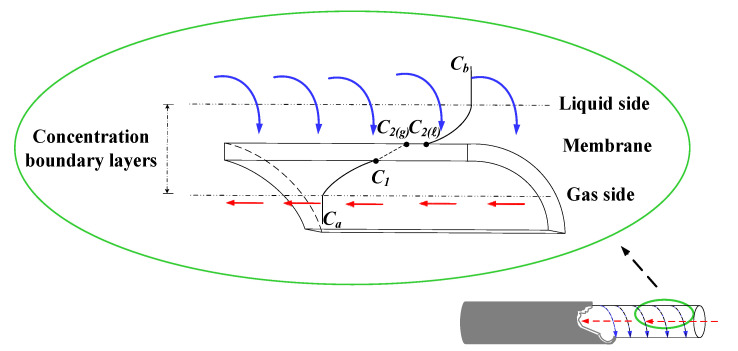
Schematic concentration profiles and boundary layers of a spiral wired annulus channel.

Mathematical modeling equations were formulated considering both diffusion and chemical reactions to calculate the CO_2_ absorption rate in the concentric circular membrane contactor module. The mass diffusion occurs in the inner side of the concentric tube and reaches the porous membrane’s mouth, while the reaction takes place on the membrane surface in the shell side of the amine solution, as schematically illustrated in Figure 3.

The isothermal diffusion-reaction process in the membrane contactor module generates the trans-membrane mass flux of CO_2_ which depends on the concentration difference across the membrane, resulting in CO_2_ absorption flux. The mass-transfer rate is controlled by the concentration boundary layers on both bulk streams, the properties of the membrane and the operating conditions. The theoretical analysis of CO_2_ absorption by using MEA was developed with the following assumptions:

(a) The system is operated at steady-state and normal pressure conditions;
(b) The porous hydrophobic membrane is not wetted by the MEA solution;
(c) The membrane material does not react with the MEA solution;
(d) Henry’s law applies to the interface between the gas phase and the liquid phase.

**Figure 4:**
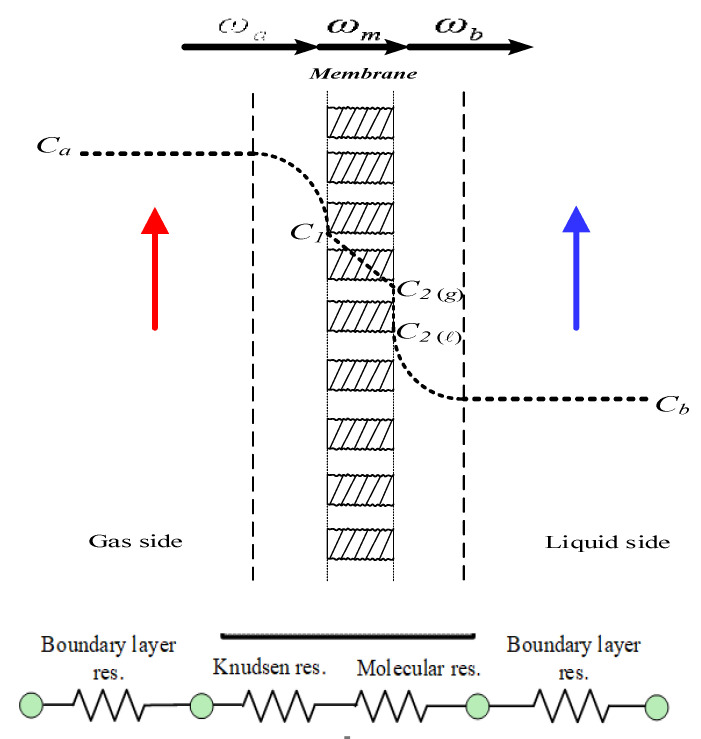
Schematic diagram of mass-transfer resistances and CO_2_ concentration variations in a gas-liquid membrane contactor.

Mass-transfer resistances in series were connected and built up across the membrane adjacent to two bulk streams, including the CO_2_ transferring to the membrane surface, generating trans-membrane flux by Knudsen diffusion and molecular diffusion, and reaching the membrane–liquid interface to be reacted by the MEA absorbent, as with the mass-transfer resistances and CO_2_ concentration variations illustrated in Figure 4. The mass-transfer rate depends only on convective mass-transfer coefficients when neglecting the bottleneck of reaction rate, and the CO_2_ concentration on the membrane–liquid interface was determined by the dimensionless Henry’s law constant $H_{c}=0.73$ [39].

The mass diffusion between both gas and liquid bulk streams and membrane surfaces, respectively, of CO_2_ was transported by the concentration driving-force gradient, as depicted below:

(1) $$\omega_{a}=k_{a}\left(C_{a}-C_{1}\right)$$

(2) $$\omega_{b}=k_{b}\left(\frac{{K^{\prime }}_{ex}C_{2\left(\ell \right)}}{H_{c}}-\frac{C_{b\left(\ell \right)}}{H_{c}}\right)$$

Application of dusty gas model [23] to the mass transfer in the membrane was considered [44], and the mass flux of CO_2_ was evaluated using a membrane permeation coefficient ($c_{m}$) and the trans-membrane saturation partial pressure differences (ΔP) [45] as

(3) $$\omega_{m}=c_{m}(P_{1}-P_{2})\frac{1}{M_{w}}={\left.c_{m}\frac{dP}{dC}\;\right|}_{C_{mean}}(C_{1}-C_{2(g)})\frac{1}{M_{w}}\;=c_{m}RT(C_{1}-\frac{{K^{\prime }}_{ex}C_{2\left(\ell \right)}}{H_{c}})\frac{1}{M_{w}}=K_{m}(C_{1}-\frac{{K^{\prime }}_{ex}C_{2\left(\ell \right)}}{H_{c}})$$

in which, $K_{m}$ is the overall mass-transfer coefficient of membrane, and the reduced equilibrium constant at T=298K  [46] and the membrane permeation coefficient [47] with the tortuosity τ=1/ε [48] were determined as

(4) $$K_{ex}^{’}=K_{ex}{[\mathrm{MEA}]/[H}^{+}],K_{ex}={[\mathrm{MEACOO}}^{-}{]\;[H}^{+}{]/[\mathrm{CO}}_{2}][\mathrm{MEA}]=1.25\times 10^{-5}$$

(5) $$c_{m}={\left(\frac{1}{c_{K}}+\frac{1}{c_{M}}\right)}^{-1}={\left\{{\left[1.064\frac{\varepsilon \;r_{p}}{\tau \delta_{m}}{\left(\frac{M_{w}}{RT_{m}}\right)}^{1/2}\right]}^{-1}+{\left[\frac{1}{{\left|Y_{m}\right|}_{\ln}}\frac{D_{m}\varepsilon }{\delta_{m}\tau }\frac{M_{w}}{RT_{m}}\right]}^{-1}\right\}}^{-1}$$

Equating the amount of mass flux in three regions transferred through the gas feed side, the membrane porous and liquid feed side was made by the conservation law as

(6) $$\omega_{i}=\omega_{a}=\omega_{m}=\omega_{b}\;i=spiral,\;empty$$

### 2.2. Concentration Polarization

The concentration polarization was controlled by the gas and liquid boundary layers in term of the concentration polarization coefficient $\gamma_{m}$. The value of the concentration polarization coefficient $\gamma_{m}$ is the extent to measuring the magnitude of mass-transfer resistances in the CO_2_/MEA absorption module. A higher value of $\gamma_{m}$ represents the absorption process with a smaller mass-transfer resistance. The undesirable influence on the mass-transfer rate was overwhelmed by disrupting the boundary layers, and thus, the absorption flux improvement with mass-transfer resistance reduction is achieved. The one-dimensional mathematical treatments were developed under steady-state operations according to the conservation of mass flux, such as in Equation (6) and as illustrated by the schematic diagram in Figure 4. Both membrane surface concentrations ($C_{1}$ and $C_{2(\ell )}$) and the convective heat-transfer coefficients ($k_{b}$) were obtained by equating Equations (1) and (3) ($\omega_{m}=\omega_{a}$) and Equations (2) and (3) ($\omega_{m}=\omega_{b}$), respectively, as follows:

(7) $$C_{a}=C_{1}+\frac{k_{m}}{k_{a}}\left(C_{1}-\frac{{K^{\prime }}_{ex}C_{2\left(\ell \right)}}{H_{c}}\right)$$

(8) $$\frac{C_{b\left(\ell \right)}}{H_{c}}=\frac{{K^{\prime }}_{ex}C_{2\left(\ell \right)}}{H_{c}}-\frac{k_{m}}{k_{b}}\left(C_{1}-\frac{{K^{\prime }}_{ex}C_{2\left(\ell \right)}}{H_{c}}\right)$$

An expression of the concentration polarization coefficient $\gamma_{m}$ was obtained by subtracting Equation (7) from Equation (8)

(9) $$\gamma_{m}=\frac{\left(C_{1}-\frac{{K^{\prime }}_{ex}C_{2\left(\ell \right)}}{H_{c}}\right)}{\left(C_{a}-\frac{C_{b\left(\ell \right)}}{H_{c}}\right)}=\frac{k_{a}k_{b}}{k_{a}k_{b}+k_{m}k_{a}+k_{m}k_{b}}$$

The calculation procedure of theoretical predictions of the mass-transfer coefficient was described as follows. First, with the given operation conditions, the mass-transfer coefficient is determined from Equations (7) and (8). Next, with the given inlet and outlet concentrations ($C_{a}$ and $C_{b}$) of both CO_2_/N_2_ gas and MEA feed streams, initial values of the concentrations on both sides of membrane surfaces $C_{1}$ (or $C_{2(\ell )}$) are estimated from Equation (7) once $C_{2(\ell )}$ (or $C_{1}$) is assumed in Equation (8). Further, the mass-transfer coefficient of the membrane is calculated from Equation (3). With this calculated value for the mass-transfer coefficient of the membrane, new values of $C_{1}$ and $C_{2(\ell )}$ are then recalculated by iterations of Equations (7) and (8) until convergence with an acceptable error of accuracy control. If the calculated values of $C_{1}$ and $C_{2(\ell )}$ deviated from the initial value, iterative calculation is continued until the last assumed values of membrane surface concentrations meet the finally calculated values.

The inner tube and lumen side of the CO_2_/MEA membrane absorption module were flowing the CO_2_/N_2_ gas feed and MEA liquid feed, respectively, as shown in Figure 3. The modeling equations of mass balances of the gas feed and liquid feed streams were derived by making the mass flux diagram presented in a finite control element under concurrent-flow and countercurrent-flow operations in Figure 2a,b, respectively, giving:

(10) $$\frac{dC_{a}}{dz}=\frac{-2\pi \;r_{i}}{q_{a}}\left[K_{m}\left(C_{1}-\frac{{K^{\prime }}_{ex}C_{2}{}_{\left(\ell \right)}}{H_{c}}\right)\right]=\frac{-2\pi r_{i}}{q_{a}}\left[K_{m}\gamma_{m}\left(C_{a}-\frac{C_{b(\ell )}}{H_{c}}\right)\right]$$

(11) $$\frac{dC_{b}}{dz}=\frac{-k_{CO_{2}}C_{b(\ell )}\pi (r_{o}^{2}-r_{i}^{2})}{q_{b}}+\frac{2\pi \;r_{i}}{q_{b}}\left[K_{m}\left(C_{1}-\frac{{K^{\prime }}_{ex}C_{2}{}_{\left(\ell \right)}}{H_{c}}\right)\right]\;=\frac{-k_{CO_{2}}C_{b(\ell )}\pi (r_{o}^{2}-r_{i}^{2})}{q_{b}}+\frac{2\pi \;r_{i}}{q_{b}}\left[K_{m}\gamma_{m}\left(C_{a}-\frac{C_{b(\ell )}}{H_{c}}\right)\right]$$

(12) $$\frac{dC_{b}}{dz}=\frac{k_{CO_{2}}C_{b(\ell )}\pi (r_{o}^{2}-r_{i}^{2})}{q_{b}}-\frac{2\pi \;r_{i}}{q_{b}}\left[K_{m}\left(C_{1}-\frac{{K^{\prime }}_{ex}C_{2}{}_{\left(\ell \right)}}{H_{c}}\right)\right]\;=\frac{k_{CO_{2}}C_{b(\ell )}\pi (r_{o}^{2}-r_{i}^{2})}{q_{b}}-\frac{2\pi \;r_{i}}{q_{b}}\left[K_{m}\gamma_{m}\left(C_{a}-\frac{C_{b(\ell )}}{H_{c}}\right)\right]$$

Equations (10) and (11) (or Equations (10) and (12)) express the mass balances derived for CO_2_ absorption in MEA absorbent under the concurrent-flow and countercurrent-flow operations, respectively, while *z* is the coordinate along with the axial flowing direction. The simultaneous ordinary equations of Equations (10) and (11) (or Equations (10) and (12)) were solved using the fourth-order Runge-Kutta method along the module’s length to determine marching solutions of the CO_2_ concentrations in both CO_2_/N_2_ and MEA feed streams, and hence, the CO_2_ absorption flux and absorption flux improvement were obtained.

### 2.3. Mass-Transfer Nhancement Factor

The spiral wired annulus channel in the concentric circular module was implemented in the MEA feed stream instead of using the device of an empty channel. The extent of mass-transfer rate enhancement was lumped into an enhancement factor [38], which is the ratio of the mass-transfer rate improvement of the spiral wired module to that of the device using an empty channel. The mass-transfer enhancement factor $\alpha^{S}$ depending on inserting spiral wires of various pitches was correlated to calculate the augmented mass-transfer coefficients in membrane contactors as follows:

(13) $$Sh^{S}=\frac{k_{b}d_{h}{}_{,spriral}}{D_{b}}=\alpha^{S}Sh_{lam}$$

For the concentric circular membrane contactor using empty channels under laminar flow, the commonly used correlation [49] is:

(14) $$Sh_{lam}=0.023\;Re^{0.8}Sc^{0.33}$$

The Sherwood number of inserting spiral wires into flow channels can be incorporated into four dimensionless groups using Buckingham’s π theorem:

(15) $$Sh^{S}=f(\frac{L_{spiral}}{d_{h,empty}},Re,Sc)$$

where $L_{spiral}$ and $d_{h,empty}$ are the equivalent length of inserting spiral wires and the hydraulic diameters of the empty channels, respectively. The enhancement factor $\alpha^{S}$ was derived from the correlation via a regression analysis for Sherwood number in the device with spiral wired annulus channel as

(16) $$\alpha^{S}=0.125\;\ln{\left(\frac{L_{spiral}}{d_{h,\;empty}}\right)}^{1.504}=\frac{Sh^{S}}{Sh_{lam}}$$

in which the correlated Sherwood numbers for the device with an empty channel are in linear uniformity with the experimental data, as referred to in Equation (14).

### 2.4. Absorption Flux Improvement

The absorption flux improvement $I_{spiral}$ was illustrated by calculating the percentage increase in the device with inserting spiral wires, based on the device of an empty channel as

(17) $$I_{spiral}^{con}\left(\%\right)=\frac{\omega_{spiral}^{con}-\omega_{empty}^{con}}{\omega_{empty}^{con}}\times 100$$

(18) $$I_{spiral}^{counter}\left(\%\right)=\frac{\omega_{spiral}^{counter}-\omega_{empty}^{con}}{\omega_{empty}^{con}}\times 100$$

(19) $$I_{empty}^{counter}\left(\%\right)=\frac{\omega_{empty}^{counter}-\omega_{empty}^{con}}{\omega_{empty}^{con}}\times 100$$

where $I_{empty}^{counter}$, $I_{spiral}^{con}$ and $I_{spiral}^{counter}$ are the absorption flux improvement for countercurrent-flow operations with empty channel, and concurrent- and countercurrent-flow operations with spiral-wired channel, respectively. Meanwhile, the subscripts *spiral* and *empty* represent the channels with/without inserting spiral wires, respectively, and the superscripts *con* and *counter* represent concurrent- and countercurrent- flow operations, respectively.

The further CO_2_ absorption flux enhancement $E_{spiral}$ in CO_2_ absorption flux by inserting spiral wires in the flow channel is calculated based on the device of the same working dimensions as in the device under countercurrent-flow operations using the device of an empty channel as follows:

(20) $$E_{spiral}=\frac{\omega_{spiral}^{counter}-\omega_{empty}^{counter}}{\omega_{empty}^{counter}}=[\frac{\left(\omega_{spiral}^{counter}-\omega_{empty}^{con})-(\omega_{empty}^{counter}-\omega_{empty}^{con}\right)}{\omega_{empty}^{con}}](\omega_{empty}^{con}/\omega_{empty}^{counter})\;=(I_{spiral}^{counter}-I_{empty}^{counter})(\omega_{empty}^{con}/\omega_{empty}^{counter})=\frac{I_{spiral}^{counter}-I_{empty}^{counter}}{1+I_{empty}^{counter}}$$

### 2.5. Power Consumption Increment

The increment in energy consumption was unavoidable due to the increased frictional loss by employing a spiral wired annulus channel in the concentric-tube membrane contactor module. The power consumption includes the involvements from both the gas side and the MEA side, which can be determined using Fanning friction factor $f_{F}$ for both laminar and turbulent flows [50]:

(21) $$H_{i}=q_{a}\;\rho_{CO{\;}_{2}}\ell w_{f,CO{\;}_{2}}+q_{b}\;\rho_{MEA}\ell w_{f,MEA}\;i=\mathrm{spiral},\;\mathrm{empty}$$

(22) $$\ell w_{f,j}=\frac{2f_{F,j}{\bar{v}}_{j}^{2}L}{d_{h,i}},\;j=CO_{2},MEA$$

The average velocity and equivalent hydraulic diameter of each flow channel were calculated as follows:

(23) $${\bar{\nu }}_{CO_{2}}=\frac{q_{a}}{\pi r_{i}^{2}},\;{\bar{\nu }}_{MEA}=\frac{q_{b}}{W_{p}(r_{o}-r_{i})}$$

(24) $$d_{h,CO_{2}}=2\;r_{i},\;d_{h,MEA}=\frac{4[W_{p}(r_{o}-r_{i})]}{2[W_{p}+(r_{o}-r_{i})]}$$

The relative extents $I_{P}$ of power consumption increment was illustrated by calculating the percentage increment in the device while inserting spiral wires, based on the device of the empty channel as

(25) $$I_{p}=\frac{H_{spiral}-H_{empty}}{H_{empty}}\times 100\%$$

where the subscripts of the spiral and empty channel represent the flow channels with and without inserting spiral wires, respectively.

### 2.6. The Design of Spiral Wired Annulus Channel

**Figure 5:**
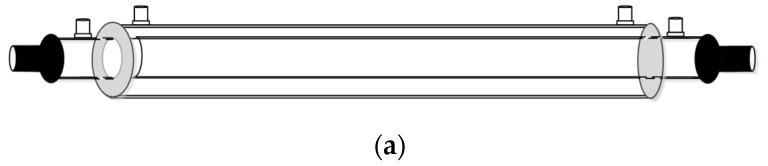

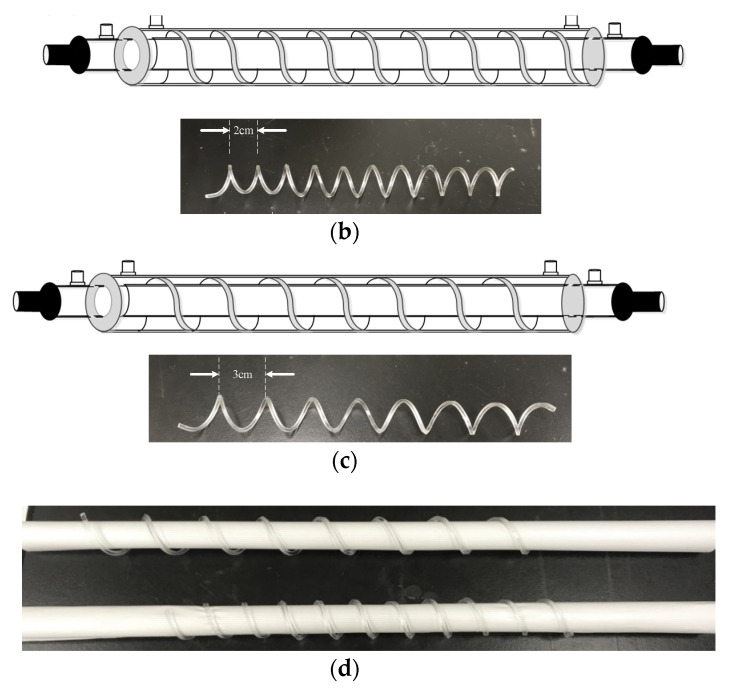
Components of concentric circular membrane contactors for the empty channel and two spiral-wire pitches of spiral wired annulus channel. (**a**) Empty channel; (**b**) 2 cm spiral-wire pitch; (**c**) 3 cm spiral-wire pitch; (**d**) Membrane tube with 2 cm and 3 cm spiral-wire pitches.

An attempt was proposed in the last two decades to augment turbulence intensity by implementing eddy promoters into the flow channel, resulting in better device performance of membrane separation processes, which destroy the concentration boundary layers on the membrane surface and come out with economic sense in terms of operation efficiency. The spiral wired annulus channel presents the advantage of reduction of concentration polarization inside the boundary layers on the membrane surface due to the productions of the turbulent behavior in enhancing a larger convective heat-transfer coefficient. Two spiral-wire pitches in the flowing channel and empty channel (without inserting spiral wire) were conducted in the experimental work, as shown in Figure 5, respectively. 

The detailed parts of the concentric circular membrane contactor module while inserting spiral wires in the flow channel are presented in Figure 5. The dimensions of the spiral-wire pitches are specified in Figure 5 for the spiral-wire pitches of 2 cm and 3 cm, respectively. The empty channel (without embedding spiral wire) is constructed by inserting an effectively 0.2 m long concentric tubular acrylic ring tube of outer diameter 1.53 cm. The acrylic helical wires were made by poly-methyl methacrylate (PMMA), and its stability testing was observed with no degradation during operating experimental runs. The inner acrylic tube was perforated up to 70% porosity by punching small circle holes of 2 mm diameter, which was wound by the hydrophobic PTFE membrane (Advantec, Japan) with a nominal pore size of 0.2 µm, a porosity of 0.72, and a thickness of 130 µm, to allow the gas diffusion through the membrane. The spiral wired annulus channel embedded helical wire is made of a 2 mm × 2 mm cross-sectional area acting as eddy promoters with spiral wire pitches of 2 cm and 3 cm, respectively, while the empty channel was wound and routed with a 0.2 mm nylon fiber on the circumference of the membrane surface on the outside of the inner tube.

## 3. Experimental Study

**Figure 6:**
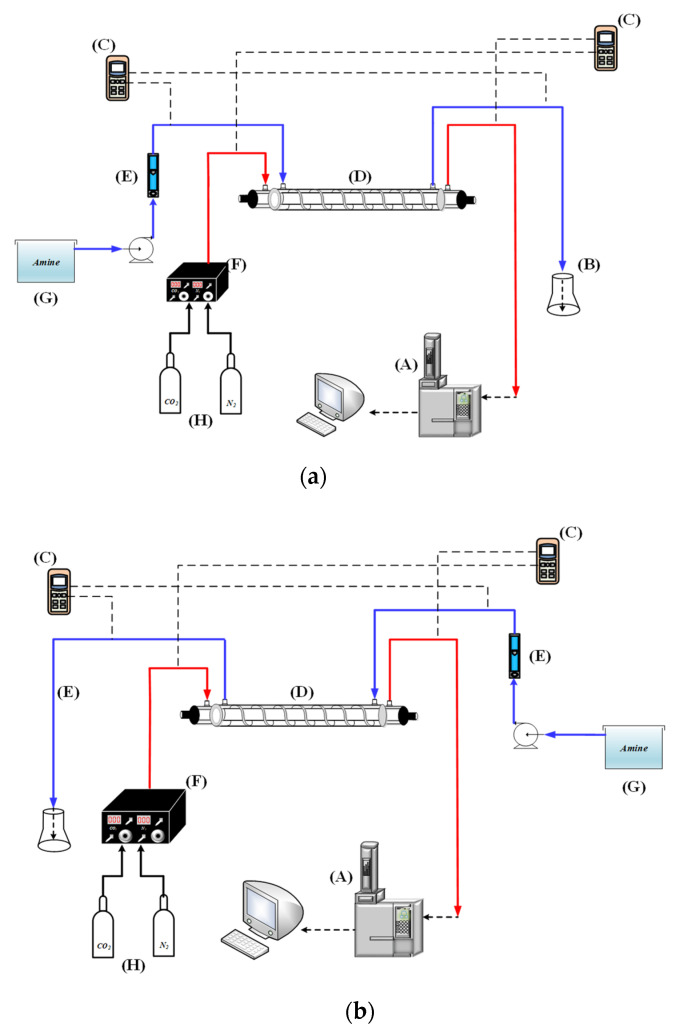
Experimental setup of spiral wired annulus channel in concentric-tube membrane module; (A) chromatograph; (B) beaker; (C) temperature indicator; (D) spiral wired concentric module; (E) flow meter; (F) mass flow controller; (G) thermostatic tank; (H) gas cylinder. (**a**) Concurrent-flow operations; (**b**) Countercurrent-flow operations.

A schematic diagram of the experimental setup of the concentric circular gas–liquid membrane contactor for CO_2_ absorption by MEA absorbent was presented as illustrated in Figure 6. The spiral wired concentric circular modules under concurrent- and countercurrent-flow operations while inserting spiral wires into the lumen side along the acrylic ring tube are illustrated in Figure 2 and Figure 5. Figure 6a,b illustrate the schematic representations of the concentric circular membrane contactor module with a spiral wired annulus channel, where the MEA solution is passing through the shell side and the gas feed is flowing through the tube side.

The aqueous MEA solution was regulated by a flow meter (MB15GH-4-1, Fong-Jei, New Taipei, Taiwan) as the liquid flowing through the lumen side from a reservoir. The experimental runs were carried out 30 wt% MEA ($5.0\times 10^{3}$ mol/m^3^) for various feed flow rates within the range of 5~10 cm^3^/s (5.0, 6.67, 8.33, 10.0 cm^3^/s). A gas mixture containing CO_2_/N_2_ introduced from the gas mixing tank (EW-06065-02, Cole Parmer Company, Illinois, USA) was regulated by using the mass flow controller (N12031501PC-540, Protec, Brooks Instrument, USA) at 5 cm^3/^s with three inlet CO_2_ concentrations of 30%, 35%, and 40%, respectively. The CO_2_ concentrations exiting in the outlet gas stream of the various operating conditions were tested and measured for comparisons by using the gas chromatography (Model HY 3000 Chromatograph, China Corporation).

The accuracy deviation [51] of the experimental results from the theoretical predictions was calculated using the following definition as:

(26) $$Er\;(\%)=\frac{1}{N_{\exp}}\sum_{i=1}^{N_{\exp}}\frac{\left|\omega_{theo,i}-\omega_{\exp,i}\right|}{\omega_{\exp,i}}\times 100$$

where *N_exp_* $\omega_{theo,i}$ and $\omega_{\exp,i}$ are the number of experimental runs, theoretical predictions, and experimental results of absorption fluxes, respectively. The accuracy deviations with two flow patterns with a 2 mm spiral-wire pitch are shown in Table 1 as an illustration. The agreement of experimental results deviated from theoretical predictions is quite good within $1.28\times 10^{-2}\leq Er\leq 3.33\times 10^{-2}$.

## 4. Results and Discussions

### 4.1. Correlated Sherwood Numbers

One may apply the Runge-Kutta numerical scheme in a marching solution procedure of Equations (10) and (11) to obtain the CO_2_ concentrations’ distributions in the CO_2_/MEA bulk streams, as well as the CO_2_ absorption flux for concurrent-flow operations, while the iterative calculation of Equations (10) and (12) can be done by a shooting strategy for the countercurrent-flow operations whilst assuming the initial guess of CO_2_ concentration at the inlet of the MEA feed stream. Comparisons were made for the CO_2_ absorption flux of modules using the spiral wired annulus channel and empty channel under both concurrent- and countercurrent-flow operations.

**Figure 7:**
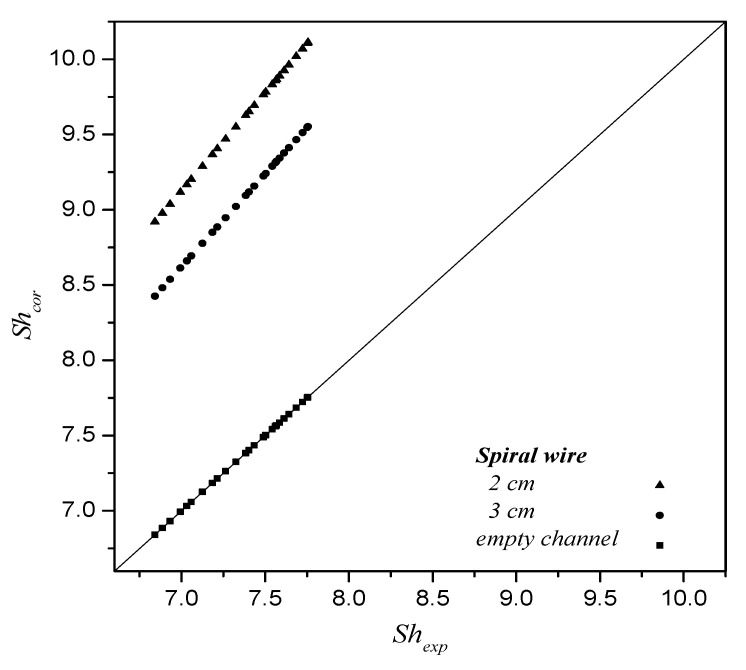
Comparison of correlated and experimental Sherwood numbers for the empty channel and spiral wired annulus channel with various spiral-wire pitches.

The mass-transfer coefficients were determined by the theoretical model and expressed in terms of Sherwood number in comparison with the experimental data, as shown in Figure 7. The correlated Sherwood numbers, as shown in Figure 7, indicate that the mass-transfer rate of the device with a spiral wired annulus channel of 2 cm spiral-wire pitch achieves a higher mass-transfer coefficient than that of the device of a 3 cm spiral-wire pitch and empty channel as well. The impact of embedding spiral wires on the mass-transfer rate enhancement is attributed to the disruption of the concentration boundary layer, and thus, the CO_2_ absorption flux was augmented due to the mass-transfer resistance reduction. Restated, a narrower pitch of the spiral wired annulus channel induces a higher turbulence intensity that results in a larger mass-transfer rate on absorption fluxes.

### 4.2. Effects of Device Parameters and Operating Conditions on Concentration Polarization

**Figure 8:**
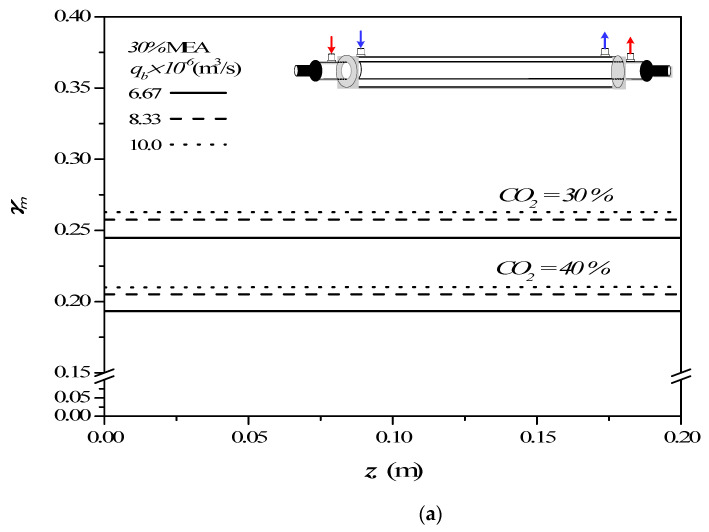

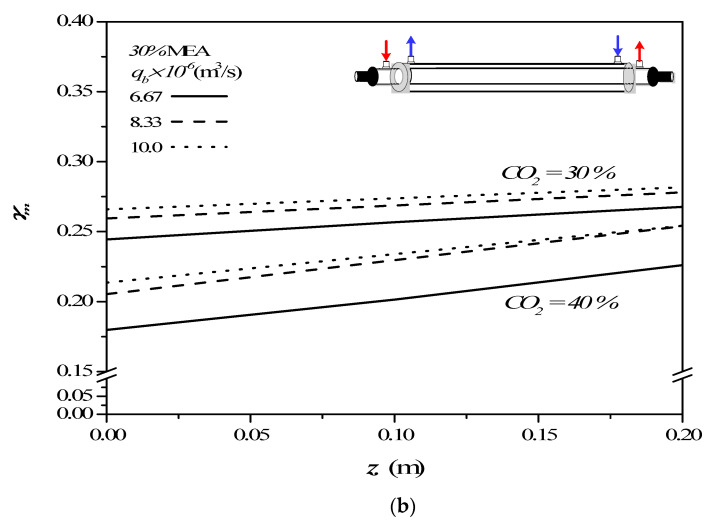
Effects of MEA flow rate and inlet feed CO_2_ concentration on $\gamma_{m}$ in the empty channel. (**a**) Concurrent-flow operations; (**b**) Countercurrent-flow operations.

The concentration polarization coefficients $\gamma_{m}$ defined in Equation (9) are an indicator of the magnitude of the mass-transfer resistance, governed by the concentration boundary layer in both gas and liquid feed streams, especially in the MEA feed side. The concentration polarization effect in the module with empty channel was examined on the value of the concentration polarization coefficient $\gamma_{m}$ as an illustration, which was demonstrated in Figure 8 along the channel direction for various MEA feed flow rates and inlet feed CO_2_ concentration.

The concentration polarization coefficients $\gamma_{m}$ were determined with various MEA feed flow rates and inlet feed CO_2_ concentrations as parameters once the predicted CO_2_ concentration distributions were obtained. The theoretical predictions of the concentration polarization coefficients $\gamma_{m}$ show that the value of $\gamma_{m}$ increases with increasing the MEA feed flow rates but with decreasing inlet feed CO_2_ concentrations. The higher the inlet CO_2_ feed concentration, a larger concentration gradient of CO_2_ on the membrane surface was produced, and hence a smaller $\gamma_{m}$ was found in Figure 8. The higher inlet feed CO_2_ concentration creates a more significant concentration polarization effect on the membrane surface. The larger inlet feed CO_2_ concentration does not accomplish a higher $\tau_{temp}\;$ value, which means the mass-transfer rate decreases when the inlet feed CO_2_ concentration is raised. This is because the higher inlet feed CO_2_ concentration does not reduce the mass-transfer resistance built up in the concentration boundary layer on the membrane surface in the MEA bulk flow. Therefore, the increased CO_2_ concentration caused by the higher inlet feed CO_2_ concentration cannot accordingly be quickly diffused to the membrane surface. Moreover, the concentration polarization coefficients $\gamma_{m}$ increase along the MEA flowing direction in concurrent-flow operations, but decrease in the reverse *z* direction of countercurrent-flow operations. Similar influences of MEA feed flow rates and inlet feed CO_2_ concentrations on concentration polarization coefficients $\gamma_{m}$ were confirmed in both countercurrent-flow and countercurrent-flow operations from Figure 8.

**Figure 9:**
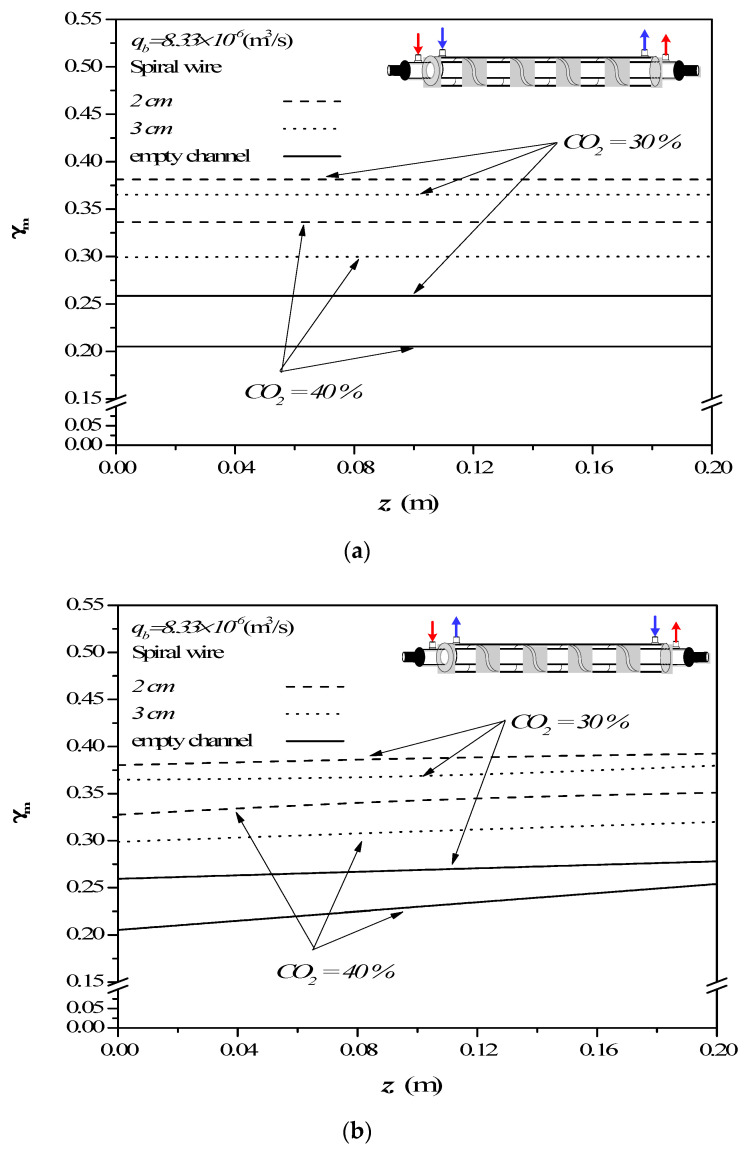
Effects of spiral-wire pitches and CO_2_ concentration on $\gamma_{m}$. (**a**) Concurrent-flow operations; (**b**) Countercurrent-flow operations.

The concentration polarization coefficients $\gamma_{m}$ is an indicator to measure the magnitude of the mass-transfer resistance, which is attributed to the higher feed flow rate and the larger turbulence intensity created by operating a spiral wired annulus channel. The absorption flux improvement was enhanced by implementing the spiral wired annulus channel in examining the value of the concentration polarization coefficient $\gamma_{m}$. The main contribution to diminishing the concentration polarization in the boundary layer on the membrane surface was accomplished by the effects of turbulent flow due to a higher MEA feed flow rate, and an eddy promotion owing to inserting spiral wires. The theoretical predictions of the concentration polarization coefficient $\gamma_{m}$ in operating the modules with inserting spiral-wire pitches of 3 cm and 2 cm under the inlet feed CO_2_ concentrations of 30% and 40%, respectively, were calculated in comparison to that of the module with the empty channel under both concurrent- and countercurrent-flow operations, as shown in Figure 9.

The turbulence intensity promotion by inserting spiral wires in both concurrent- and countercurrent-flow operations aimed to shrink concentration polarization layers and diminish the mass-transfer resistance as well, whereby the absorption flux is enhanced. The results show that the value of $\gamma_{m}$ for the 2 cm pitch of the spiral wired channel is larger than those of the 3 cm pitch of the spiral wired channel, as well as the module with an empty channel, which means the operating 2 cm pitch of the spiral wired channel resulted in a lesser mass-transfer resistance for CO_2_ absorption. Restated, inserting spiral wires in the flow channel is a positive influence on the eddy promotion, and the smaller spiral-wire pitch generates a higher convective mass-transfer coefficient, which comes out with a higher $\gamma_{m}$ value and a higher absorption flux.

### 4.3. CO_2_ Absorption Flux Enhancement by Embedding Spiral Wires

**Figure 10:**
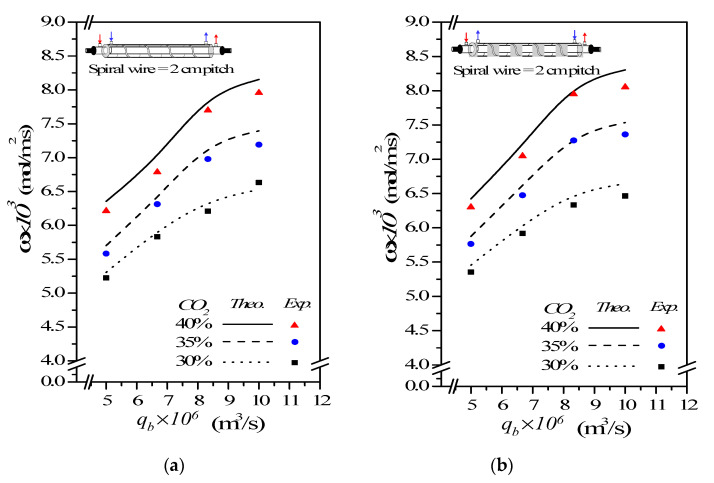
Effects of MEA flow rate and inlet CO_2_ feed concentration on CO_2_ absorption flux. (**a**) Concurrent-flow operations; (**b**) Countercurrent-flow operations.

**Figure 11:**
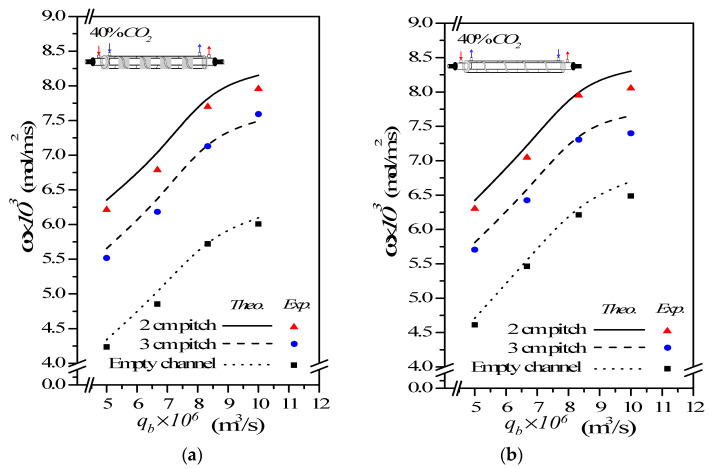
Effects of MEA flow rate and spiral-wire pitch on CO_2_ absorption flux. (**a**) Concurrent-flow operations; (**b**) Countercurrent-flow operations.

This study has shown that the CO_2_ absorption flux for the module with embedding spiral-wire pitches of 2 cm and 3 cm in both concurrent- and countercurrent-flow operations, as shown in Figure 10 and Figure 11 including both experimental results and theoretical predictions, respectively.

(**a**) Concurrent-flow operations (**b**) Countercurrent-flow operations

In general, the CO_2_ absorption flux by embedding spiral wires is more noteworthy in countercurrent-flow operations than that in concurrent-flow operations. A larger concentration gradient achieved between gas and liquid in countercurrent-flow operations with respect to concurrent-flow operations results in a higher device performance on absorption flux. As expected, either the increase of both MEA feed flow rate and inlet feed CO_2_ concentration or the decrease of the spiral-wire pitch yields a higher absorption flux.

The theoretical predictions of the CO_2_ absorption flux improvement $I_{spiral}$ for various MEA feed flow rates, inlet feed CO_2_ concentrations and spiral-wire pitches under concurrent- and countercurrent-flow operations are summarized in Table 2; Table 3, respectively.

A relative increment of absorption flux improvement $I_{spiral}$ was calculated in comparison of the absorption flux in the module with spiral wired annulus channels to that of the empty channel in concurrent-flow operations. It is also seen from Table 2 and Table 3 that the order of the CO_2_ absorption flux and CO_2_ absorption flux improvement for the module embedding spiral wires is 2 cm pitch > 3 cm pitch and countercurrent-flow operations > concurrent-flow operations. The results show that the maximum absorption flux improvement up to 47.93% is obtained as compared to that in the empty channel device. Overall, the CO_2_ absorption flux augmented by inserting spiral wires is more substantial in countercurrent-flow operations than that in concurrent-flow operations. Inserting spiral wires into flow channel demonstrates a great potential to improve significantly the absorption flux, and then, the absorption flux improvement in gas/liquid membrane contactors as well.

### 4.4. Further CO_2_ Absorption Flux Enhancement

The further absorption flux enhancement is accomplished if there are various spiral-wire pitches that are embedded into MEA feed stream for increasing the convective mass-transfer coefficient, which results in the turbulence intensity increment. A maximum 47.93% absorption flux improvement is achieved with spiral wired channel rather than the same device of empty channel for 2mm spiral-wire pitch and countercurrent-flow operations for instance, as seen in Table 4. Moreover, the further absorption flux enhancement of the module with spiral wired channel increases with increasing inlet feed CO_2_ concentration but decreasing with the spiral-wire pitches and MEA feed flow rate.

### 4.5. Power Consumption Increment

**Figure 12:**
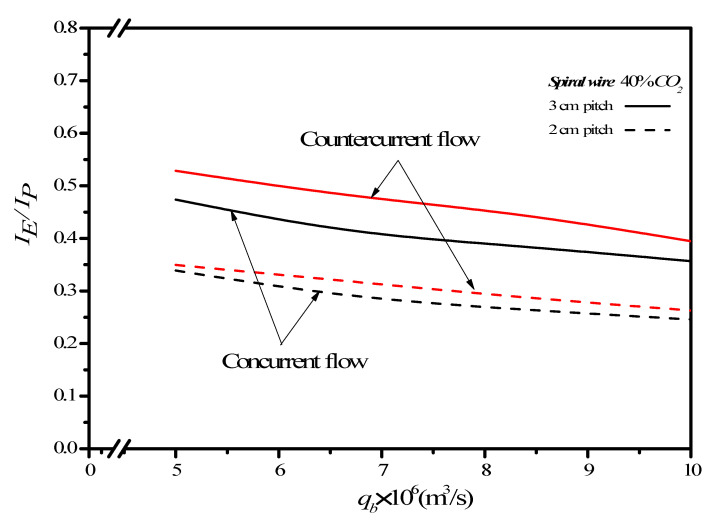
Effects of MEA feed flow rate and spiral-wire pitch on $I_{E}/I_{P}$.

Inserting spiral wires acting as turbulence promoters confronts two conflict effects of the desirable absorption flux improvement and the undesirable power consumption increment, which exists an indicator of economic viewpoint in making the suitable selection. Concerning the compensation of the CO_2_ absorption flux improvement due to friction losses increased by inserting spiral wires in the MEA feed channel, the effects of spiral-wire pitches and MEA flow rates on the ratio $I_{E}/I_{P}$ of CO_2_ absorption flux improvement to power consumption increment are shown in Figure 12. The higher the inlet feed CO_2_ concentration and the smaller spiral-wire pitch give the higher $I_{E}/I_{P}$ value. Restated, the percentage increment of absorption flux improvement is higher than the percentage increment of energy consumption. The increase of the MEA feed flow rate yields a lower ratio of $I_{E}/I_{P}$ and reaches an insignificant change for MEA feed flow rate being larger than $8.33\times 10^{-6}\;$ m^3^/s. One found that the effectiveness of inserting 3 mm spiral-wire pitch are all higher than that of 2 mm spiral-wire pitch under the same operation type. The comparison reveals that though a higher absorption flux improvement associated with a higher power consumption increment, and thus, the ratio of $I_{E}/I_{P}$ is not absolute going larger, which implies that increase of the CO_2_ absorption flux cannot compensate the increase of power consumption by increasing the MEA feed rate. In other words, the countercurrent-flow operation can utilize energy efficiency to increase CO_2_ absorption flux more effectively than that in the concurrent-flow operation regarding to the economic consideration.

## 5. Conclusions

Promoting turbulence intensity in a concentric circular gas-liquid PTFE membrane contactor for CO_2_ absorption was designed by embedding spiral wires into the concentric annulus channel, and the mathematical modeling was developed theoretically and validated experimentally. The results has demonstrated its technical and economic feasibility in terms of the ratio of $I_{E}/I_{P}$ and obtaining up to 47.93% absorption flux enhancement by implementing spiral wired annulus channel. The value of this study are twofold:

(1) to propose a new device of inserting spiral wires including the desirable effect in raising the turbulence intensity by an alternative strategy on the CO_2_ absorption in MEA absorbent through concentric circular membrane contactor;

(2) to present graphically the concentration polarization coefficient and CO_2_ absorption flux with MEA feed flow rates, inlet feed CO_2_ concentrations and spiral-wire pitches as parameters under both concurrent- and countercurrent-flow operations.

Furthermore, an expression of Sherwood number was obtained to correlate the mass-transfer coefficient of the gas/liquid membrane contactor module with embedding spiral wired annulus channel.

## Figures and Tables

**Table 1:** The accuracy deviation between theoretical predictions and experimental results.

$C_{in}$$q_{b}\times 10^{6}$ (%) m^3^/s		Concurrent Flow			Countercurrent Flow		
		$\omega_{\exp}^{con}\times 10^{3}$	$\omega_{theo}^{con}\times 10^{3}$	Er(% )	$\omega_{\exp}^{counter}\times 10^{3}$	$\omega_{theo}^{counter}\times 10^{3}$	Er(% )
**30**	5.0	5.23	5.31	1.53	5.35	5.45	1.83
	6.67	5.83	5.92	1.51	5.92	6.05	2.14
	8.33	6.21	6.37	2.50	6.33	6.51	2.78
	10.0	6.63	6.53	2.08	6.46	6.65	1.60
40	5.0	5.58	5.70	2.10	5.76	5.87	1.87
	6.67	6.31	6.40	1.41	6.47	6.61	2.12
	8.33	6.98	7.22	3.33	7.27	7.37	1.28
	10.0	7.19	7.39	2.75	7.36	7.54	2.29
45	5.0	6.21	6.35	2.22	6.30	6.42	1.88
	6.67	6.79	6.99	2.86	7.05	7.20	2.06
	8.33	7.70	7.94	3.02	7.95	8.12	2.05
	10.0	7.96	8.15	2.40	8.05	8.30	3.03

**Table 2:** Effects of spiral-wire pitches on absorption flux improvements.

$C_{in}$$q_{b}\times 10^{6}$ (%) (m^3^ s^−1^)		Concurrent-Flow Operations (mol m^−2^ s^−1^)				
		Empty Channel	2 cm Spiral Wire		3 cm Spiral Wire	
		$\omega_{theo}^{con}\times 10^{3}$	$\omega_{theo}^{con}\times 10^{3}$	$I_{spiral}^{con}$ (%)	$\omega_{theo}^{con}\times 10^{3}$	$I_{spiral}^{con}$ (%)
**30**	5.0	3.93	5.31	35.18	4.77	21.61
	6.67	4.46	5.92	32.84	5.30	18.89
	8.33	4.90	6.37	30.06	5.67	15.78
	10.0	5.07	6.53	28.80	5.87	15.71
**35**	5.0	4.08	5.70	39.87	5.11	25.41
	6.67	4.68	6.40	36.76	5.79	23.62
	8.33	5.42	7.22	33.09	6.59	21.43
	10.0	5.62	7.39	31.60	6.77	20.42
40	5.0	4.34	6.35	46.45	5.66	30.35
	6.67	5.01	6.99	39.42	6.33	26.22
	8.33	5.82	7.94	36.31	7.26	24.74
	10.0	6.10	8.15	33.71	7.49	22.87

**Table 3:** Effects of spiral-wire pitches on absorption flux improvements.

$C_{in}$$q_{b}\times 10^{6}$ (%) (m^3^ s^−1^)		Countercurrent-Flow Operations (mol m^−2^ s^−1^)				
		Empty Channel	2 cm Spiral Wire		3 cm Spiral Wire	
		$\omega_{theo}^{counter}\times 10^{3}$	$\omega_{theo}^{counter}\times 10^{3}$	$I_{spiral}^{counter}$ **(%)**	$\omega_{theo}^{counter}\times 10^{3}$	$I_{spiral}^{counter}$ **(%)**
**30**	5.0	4.12	5.45	38.68	4.92	25.19
	6.67	4.67	6.05	35.65	5.43	21.75
	8.33	5.15	6.51	32.86	5.79	18.16
	10.0	5.28	6.65	31.16	5.92	16.77
**35**	5.0	4.38	5.87	43.87	5.27	29.17
	6.67	5.15	6.61	41.24	6.00	28.21
	8.33	5.86	7.37	35.98	6.76	23.06
	10.0	6.06	7.54	34.16	6.95	21.57
40	5.0	4.71	6.42	47.93	5.81	33.87
	6.67	5.55	7.20	43.71	6.55	30.74
	8.33	6.40	8.12	39.52	7.48	28.52
	10.0	6.70	8.30	36.07	7.65	25.41

**Table 4:** Theoretical predictions of absorption flux improvements and further absorption flux.enhancement with inserting spiral wires.

$C_{in}$$q_{b}\times 10^{6}$ (%) m^3^/s		Countercurrent-Flow Operations				
		Empty Channel	2 cm Spiral Wire		3 cm Spiral Wire	
		$I_{empty}^{counter}$ (%)	$E_{spiral}$ (%)	$I_{spiral}^{counter}$ (%)	$E_{spiral}$ (%)	$I_{spiral}^{counter}$ (%)
**30**	5.0	4.85	32.27	38.68	20.24	25.19
	6.67	4.71	29.27	35.65	16.27	21.75
	8.33	5.10	26.41	32.86	12.43	18.16
	10.0	4.14	25.95	31.16	12.13	16.77
**35**	5.0	7.35	34.01	43.87	20.33	29.17
	6.67	10.04	28.35	41.24	16.51	28.21
	8.33	8.19	25.69	35.98	13.75	23.06
	10.0	7.83	24.42	34.16	12.74	21.57
40	5.0	8.53	36.30	47.93	24.07	33.87
	6.67	10.78	29.73	43.71	19.61	30.74
	8.33	9.97	26.87	39.52	16.87	28.52
	10.0	9.84	23.88	36.07	14.18	25.41

## Abbreviations

C	Concentration (mol m^−3^)
$C_{mean}$	Mean value of C (mol m^−3^)
$c_{k}$	Membrane coefficient based on the Knudsen diffusion model (mol m^−2^ Pa^−1^ s^−1^)
$c_{M}$	Membrane coefficient based on the molecular diffusion model (kg m^−2^ Pa^−1^ s^−1^)
$c_{m}$	Membrane permeation coefficient (mol m^−2^ Pa^−1^ s^−1^)
$D_{b}$	Diffusion coefficient of CO_2_ in MEA (m^2^ s^−1^)
$d_{h,i}$	Equivalent hydraulic diameter of channel (*m*), *i* = spiral, empty
Er	Accuracy deviation of experimental results from the theoretical predictions
E	absorption flux enhancement
$f_{F}$	Fanning friction factor
$H_{C}$	Dimensionless Henry’s constant
$H_{i}$	Hydraulic dissipate energy (J kg^−1^), *i* = spiral, empty
$I_{E}$	Absorption flux enhancement
$I_{P}$	Power consumption relative index
$\omega_{i}$	Molar flux (mol m^−2^ s^−1^)
$k_{a}$	Mass-transfer coefficient in the gas feed stream (m s^−1^)
$k_{b}$	Mass-transfer coefficient in the liquid absorbent side (m s^−1^)
$K_{ex}$	Equilibrium constant
${K^{\prime }}_{ex}$	Reduced equilibrium constant
$K_{m}$	Overall mass-transfer coefficient of membrane (m s^−1^)
$\ell w_{f,j}$	Friction loss (J kg^−1^), *j* = CO_2_, MEA
L	Channel length (m)
$L_{spiral}$	Length of spiral wired channel (m)
$M_{W}$	Average molecular weight of CO_2_ and N_2_ gas mixture (kg mol^−1^)
*N_exp_*	Number of experimental measurements
$P_{1}$	Saturation vapor pressure in the gas feed flow side (Pa)
$P_{2}$	Saturation vapor pressure in the liquid absorbent flow side (Pa)
$q_{a}$	Volumetric flow rate of the gas feed stream (m^3^ s^−1^)
$q_{b}$	Volumetric flow rate of the MEA absorbent side (m^3^ s^−1^)
R	Gas constant (8.314 J mol^−1^ K^−1^)
*Re*	Reynolds number
$r_{i}$	Radius of inner tube (m)
$r_{o}$	Radius of shell (m)
*r_p_*	Membrane pore radius (m)
*Sc*	Dimensionless Schmidt number
*Sh^S^*	Enhanced dimensionless Sherwood number
$Sh_{lam}$	Dimensionless Sherwood number for laminar flow
*W_p_*	Pitch width (m)
ω	Absorption flux (mol m^−2^ s^−1^)
${\left|Y_{m}\right|}_{\ell n}$	Natural log mean CO_2_ mole fraction in the membrane
z	Axial coordinate along the flow direction (m)
*Greek letters*	
$\alpha^{S}$	Mass-transfer enhancement factor
β	Aspect ratio of the channel
$\delta_{m}$	Thickness of membrane (µm)
ε	Membrane porosity
$\bar{\nu }$	Average velocity (m s^−1^)
$\rho_{i}$	Density (kg m^−3^), *i* = CO_2_, MEA
$\gamma_{m}$	Concentration polarization coefficients
*Subscripts*	
1	Membrane surface on MEA side
2(l)	Liquid phase on membrane surface on MEA side
2 ( g)	Gas phase on membrane surface on MEA side
*a*	The gas feed flow channel
*b*	The liquid absorbent flow channel
*cal*	Calculated results
*spiral*	Inserting spiral wires as promoters
*empty*	Empty channel
*exp*	Experimental results
*in*	Inlet
*out*	Outlet
*theo*	Theoretical predictions
*Superscripts*	
*con*	Concurrent-flow operations
counter	Countercurrent-flow operations
